# Visiting Richard Serra’s “Promenade” sculpture improves postural control and judgment of subjective visual vertical

**DOI:** 10.3389/fpsyg.2014.01349

**Published:** 2014-12-12

**Authors:** Zoï Kapoula, Alexandre Lang, Thanh-Thuan Lê, Marie-Sarah Adenis, Qing Yang, Gabi Lipede, Marine Vernet

**Affiliations:** IRIS Team, Physiopathologie de la Vision et Motricité Binoculaire, CNRS FR3636, UFR Biomédicale, Université Paris Descartes, Paris, France

**Keywords:** art, posture, sculpture, subjective vertical, eye movements

## Abstract

Body sway while maintaining an upright quiet stance reflects an active process of balance based on the integration of visual, vestibular, somatosensory, and proprioceptive inputs. Richard Serra’s *Promenade* sculpture featured in the 2008 *Monumenta* exhibition at the Grand Palais in Paris, France is herein hypothesized to have stimulated the body’s vertical and longitudinal axes as it showcased five monumental rectangular solids pitched at a 1.69^°^ angle. Using computerized dynamic posturography we measured the body sway of 23 visitors when fixating a cross, or when observing the artwork (fixating it or actively exploring it with eye movements) before and after walking around and alongside the sculpture (i.e., before and after a *promenade*). A first fixation at the sculpture increased medio-lateral stability (in terms of spectral power of body sway). Eye movement exploration in the depth of the sculpture increased antero-posterior stability [in terms of spectral power and canceling time (CT) of body sway] at the expense of medio-lateral stability (in terms of CT). Moreover, a medio-lateral instability associated with eye movement exploration before the *promenade* (in terms of body sway *sensu stricto*) was canceled after the *promenade*. Finally, the overall medio-lateral stability (in terms of spectral power) increased after the *promenade*. Fourteen additional visitors were asked to stand in a dark room and adjust a luminous line to what they considered to be the earth-vertical axis. The *promenade* executed within the sculpted environment afforded by Serra’s monumental statuary works resulted in significantly improved performances on the subjective visual vertical test. We attribute these effects to the sculpted environment provided by the exhibition which may have acted as a kind of physiologic “training ground” thereby improving the visitors’ overall sense of visual perspective, equilibrium, and gravity.

## INTRODUCTION

Neuroaesthetics is an innovative and rapidly expanding research field, which currently champions non-invasive neuroimaging techniques as the research methodology of choice in the investigation of the neural basis of the cognitive and affective processes triggered during an aesthetic episode. These neuroimaging studies are principally directed toward the cortical and sub-cortical activations associated with producing or viewing pictorial art, listening to music, etc. Such studies revealed that various brain areas, such as areas belonging to the reward system (e.g., orbitofrontal cortex) or to emotional processing (e.g., amygdala, insula), areas related to high-level cognitive processes (e.g., prefrontal cortex), participate in aesthetics experience (e.g., Di Dio et al., 2007; Di Dio and Gallese, 2009; Brown et al., 2011; Ishizu and Zeki, 2011). Interestingly, in line with theories of embodied cognition, several studies revealed that artworks also impact human sensorimotor system. For instance, parts of occipito-temporal and parietal areas important for observing actions as well as sensorimotor cortex are particularly activated when observing aesthetical dance movements (Calvo-Merino et al., 2008; Cross et al., 2011). Beyond the observation of real aesthetical movements, artworks conveying a sense of motion also impact the sensorimotor system. For example, the motion-sensitive brain area MT^+^ is more activated when observing abstract paintings with implied motion than abstract paintings with little motion impression, but only in observers with prior experience viewing those kinds of paintings (Kim and Blake, 2007). Finally, an EEG study revealed that observation of artworks by Lucio Fontana made of cuts on canvas evoked mu rhythm suppression, which is a typical neurophysiologic marker of movement initiation (Umilta et al., 2012).

Beyond these brain responses indicating that artworks’ observers can simulate creative processes, artworks can also impact the full body’s physiology. To evaluate such impact, posturography emerges as a field capable of providing novel insights into creative ideation, artistic creation, and aesthetic experience. By providing empirical data for theories of embodied cognition, posturography can potentially span multiple interrelated disciplines and bridge the gap between seemingly distinctive, physiology-related disciplines with their specialized and mutually exclusive focus on brain and body. Posturography quantifies aspects of postural control peculiar to upright quiet stance and it does so in a non-invasive manner across relatively short time intervals. It provides valuable information concerning the central nervous system’s ability to integrate multiple inputs (visual, vestibular, cutaneous, and muscle proprioceptive) and to generate muscular responses adapted as corrective torque by way of a feedback control system. The body is never perfectly still but is rather constantly in motion and physiologic body sway reflects these active processes. Posturography is mainly used in neurophysiology as a tool in the diagnosis and follow-up of patients suffering from balance and equilibrium disorders. Recently, two teams inaugurated this new field in aesthetics by questioning the effects of depicted body movements (Nather et al., 2010) and of pictorial depth (Kapoula et al., 2011) on body sway. Nather et al. (2010) showed that participants exhibited significantly greater body sway when observing a picture of a Degas’ sculpture of a dancing ballerina than when observing a picture of a Degas’ sculpture of a static ballerina, demonstrating that images of body movement internally generate unconscious body oscillations. Kapoula et al. (2011) showed that a pictorial representation of depth in the visual field increased body sway in much the same way that the perception of real physical depth does. This effect was, however, a function of the painting used: two abstract paintings by Maria Helena Vieira Da Silva (*Egypt*, 1948; *O quarto cinzento*, 1950) were tested in this study. These two paintings exhibited various formal and stylistic similarities and produced an equally vivid sense of depth on behalf of the observing subjects; however, only the second painting was able to modulate body sway. It was suggested that highly salient perspective constructions and other ingenious visual features of paintings can literally move the body, impacting body sway in a unique manner.

The present study pursues this innovative posturographic approach further by analyzing Richard Serra’s *Promenade* sculpture, which was featured in the context of the 2008 *Monumenta* exhibition held at the Grand Palais in Paris, France. This study was conducted *in situ* and is truly unique in terms of recent experimental investigations into art and aesthetics in so far as it deals with sculpture, a fine art which has been the object of comparatively few empirical investigations.

The first hypothesis we made was that, as with other aesthetic stimuli displaying a strong sense of depth and movement, the mere observation of the sculpture would impact postural control. Such impact could be observed in either medio-lateral or antero-posterior direction as the sculpture involved strong depth (alignment of monumental solids in depth) and lateral (tilted monumental solids) components. Moreover, the effects were expected to be subtle, as the postural parameters would remain within the normal range for healthy young individuals.

In addition, artworks are never passively observed; rather, eye movement exploration is expected to participate in aesthetics experience. For instance, illusory movements while observing artwork from the Op Art movement could emerge from an instable eye movement behavior made of numerous small saccades (Zanker et al., 2003; Zanker and Walker, 2004). Such small, or, to a greater extent, larger saccades, probably involving vergence components as well, can in turn impact postural control (Kapoula et al., 2014). Indeed, laboratory studies revealed that vergence state modulates postural parameters (Kapoula and Le, 2006; Le and Kapoula, 2008; Matheron et al., 2008) and that vergence movements have a positive impact on posture (Kapoula et al., 2013). Thus, the second hypothesis we made in this study was that actively exploring the sculpture with eye movements would modulate the postural control.

Finally, we took advantage of our *in situ* experimental configuration allowing us to test our third hypothesis, according to which a real visit of the exhibition, including walking around and alongside the sculpture while listening to information about the exhibition, stimulating physical, cognitive, and emotional processes, would have a long-term impact on the postural control.

In addition to posturography (Experiment 1), this study also introduces the use of the subjective visual vertical (SVV) test (Experiment 2), widely used since 1970 (Friedmann, 1970) in the diagnosis of disequilibrium and vestibular disorders. Here, this diagnostic tool was used to determine the impact of Serra’s sculpture on the accuracy with which visitors were able to make apparent verticality judgments. Our forth hypothesis was that changes in postural control after visiting the exhibition could be paralleled by changes in SVV evaluation.

This experimental approach applies a novel methodology. The artwork being a unique stimulus within a unique museum environment, the results can only be compared to a basic measure (fixation of a cross, run before the visit of the exhibition). This study focuses on demonstrating the effect of this unique stimulus. This, however, does not imply that ordinary objects with similar physical properties would not lead to similar physiologic impact.

## EXPERIMENT 1: POSTUROGRAPHY

### MATERIALS AND METHODS

#### Ethics statement

The investigation adhered to the tenets of the Declaration of Helsinki and was approved by the local ethics committee for human experimentation, CPP Ile de France II (No: 07035, Hôpital Necker in Paris). Written consent approved by the committee was obtained from all subjects after the nature of the experiment had been explained.

#### The artwork

**FIGURE 1:**
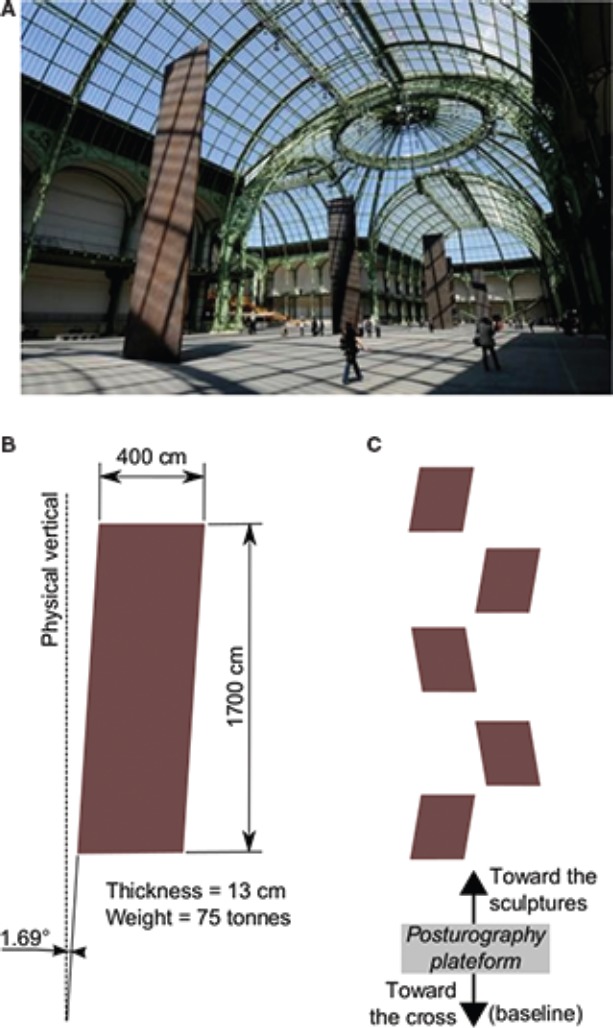
**Illustration of the artwork stimuli. (A)** Cliché from Didier Plowy of Monumenta 2008, Richard Serra’s *A stroll in the Nave*, © Didier Plowy/RMNGP. **(B)** Dimensions of the sculptures. **(C)** Illustration of the experimental configuration including the sculptures and location of the posturography platform.

The primary stimulus employed in this study consisted of Richard Serra’s *Promenade* sculpture featured in the 2008 *Monumenta* exhibition held at the Grand Palais in Paris, France (Monumenta: A New Look at the Grand Palais, n.d.). *Monumenta* is an annual exhibition which calls upon contemporary artists to showcase artworks specially designed to accommodate the Grand Palais’ 13,500 m^2^ nave of filigreed iron and glass. In 2008, the leading American artist, Richard Serra accepted the *Monumenta* challenge (tribeca75tv, 2008). Serra is highly adept at working on a large scale—whether within the confines of extensive interior spaces or in other outdoor exhibition venues. The artist created five massive, sculpted rectangular solids, which constitute his *Promenade* sculpture, and adapted their construction to the Grand Palais’ unusually spacious exhibition space. Serra’s proposed *Promenade* sculpture dealt principally with formal themes of verticality and the rectangular solids’ transverse axis constituted the works’ rhythmic counterpart. The sculptures can be minimally reduced to six determining physical qualities (height = 1700 cm, width = 400 cm, thickness = 13 cm, weight = 75 metric tons, tilt = 1.69^°^, distance between any two consecutive steal beams = 28 m). Their height was determined in relation to the Grand Palais’ arching, cruciform glass nave (rising to a height of 45 m) and they were spread across the full length of the nave. The installation of Serra’s gigantic steel slabs was determined by calculating their respective alignment according to a rhythmic sequence of asymmetrical inclines at a 1.69^°^ angle (see Figure 1).

During the exhibition, observers typically walked around and alongside the steel beams, taking in multiple viewpoints. Such behavior constitutes a unique aesthetic experience whose underline physiologic correlates form the object of this study. Indeed, one’s sense of gravity as well as both the visual and vestibular systems are challenged by Serra’s *Promenade* sculpture. We therefore designed a set of physiologic measures consisting of posturography (Experiment 1) and the SVV test (Experiment 2) in order to assess the impact of this artwork on the physiology of the observer.

#### Participants

Twenty-three young adults (26.1 ± 6.1 years) volunteered to take part in this experiment. Some of the participants came to the exhibition in response to an ad posted on a student mailing list and approached the experimenters at the entrance of the museum, while other participants were recruited directly at the entrance of the museum. None of the participants knew the hypotheses of the study.

#### Posturography apparatus

Postural stability was measured using a posturography apparatus which consists of two dynamometric soles (TechnoConcept, Céreste, France), one for each foot. The excursions of the center of pressure (CoP) were measured during 51.2 s in each condition; the equipment contained an analog-to-digital converter of 16 bits. The sampling frequency of the CoP was 40 Hz. The duration of posturography recording is not standardized. While short durations (typically 30 s) are commonly used in clinics, longer durations might allow more discriminative power on non-linear parameters (Schubert et al., 2012a,b). Our 51.2-s recording is thus a compromise between short recordings avoiding the occurrence of transient and particular events due to postural changes and consequently deteriorating measures related to sway area, and long recordings allowing a better accuracy in time-frequency analysis.

#### Procedure

The participants were placed on the posturography platform along the central axis of the Grand Palais at 15 m from the first plate (see Figure 1C). Throughout all of the posturography tests, participants stood on the posturography platform while assuming an upright position with the feet placed side-by-side forming a 30^°^ angle and the heels positioned 4 cm apart. The participants were required to maintain a quiet stance by holding their arms at their sides while breathing normally and refraining from both speaking and clenching their teeth. The five following conditions were run.

– Condition 1 (basic): fixating a cross
  When the participant entered the nave, before she/he had the opportunity to see the artwork, a 51.2-s posturography recording session was performed with the participant fixating the center of a cross (30 cm × 30 cm) located 30 m in front of her/him while turning her/his back away from the artwork.
– Condition 2: fixating the sculpture
  Immediately after the basic condition, the participant performed a 180^°^ turn in order to face the sculpture directly. She/he was then instructed to fixate the upper right edge of the last sculpture as precisely as possible. Again, the posturography was recorded during 51.2 s.
– Condition 3: eye navigation in depth along the sculptures’ transverse plane
  In the next condition, participants were asked to fixate back and forth successively each sculpture from the farthest to the nearest sculpture repeatedly during the allotted 51.2 s recording period. Oculomotor behaviors of this kind necessitate saccade–vergence eye movements, which are a commonly observed class of eye movements executed during visual explorations of the environment (Enright, 1984; Collewijn et al., 1995; Yang et al., 2002).
  The participants were then asked to walk freely both alongside and around the sculptures, thereby assuring that the subjects took in multiple points of view as would likely be the case had they freely visited the exhibition under normal circumstances. During this stage, they were listening to the exhibition audio-guide (tracks 102,103,104). The total duration of the *promenade* was about 1 h.
– Conditions 4 and 5 (post visit testing): fixation and eye navigation
  Following their *promenade*, posturography was performed under the same conditions as those followed in conditions 2 and 3.

#### Data analysis

Data were analyzed using the following postural parameters: (1) standard deviation of medio-lateral and antero-posterior body sway (SDx and SDy); (2) surface of the CoP excursions, measured with the confidence ellipse including 90% of the CoP positions which were sampled by eliminating the extreme points (Takagi et al., 1985); (3) variance of speed.

A wavelet non-linear analysis using Morlet waves (Framiral, n.d.) was applied to CoP displacements in order to elaborate a time-frequency chart of body sway (Dumistrescu and Lacour, 2006; Bernard-Demanze et al., 2009). Such analysis allows revealing temporal fluctuations in the body sway spectrum. From this analysis, several parameters were extracted for both medio-lateral and antero-posterior body sway and for three frequency bands (F1: 0.05–0.5 Hz; F2: 0.5–1.5 Hz; F3: higher than 1.5 Hz): (1) spectral power (Px and Py for F1, F2, and F3); (2) canceling time (CT) (CTx and CTy for F1, F2, and F3); as well as (3) a global postural instability index (PII). The hypothetical physiologic significance of the spectral power (Px and Py) of different bands is as follows: 0–0.5 Hz visual–vestibular (Naschner, 1979; Kohen-Raz et al., 1996; Paillard et al., 2002), 0.5–1.5 Hz cerebellar (Paillard et al., 2002), >1.5 Hz reflexive loops (Lacour et al., 2008; Bernard-Demanze et al., 2009). As a rule, power in the higher band (F3) is minimal in healthy subjects during quiet standing, but it can nevertheless be non-negligible in the elderly and in the presence of a postural pathology, or in dynamic postural conditions (Bernard-Demanze et al., 2009). The CT is the total time during which the spectral power of the body sway for a specific frequency band is canceled by the posture control mechanisms; the longer the CT of a given frequency band, the better the postural control (Dumistrescu and Lacour, 2006; Bernard-Demanze et al., 2009). The fact that, over a period of time, a certain frequency’s power is reduced to 0 demonstrates that the postural control system has been successfully engaged given that the overall entropy of the sway has been reduced. Unlike most healthy subjects who do exhibit these zero power instances in their postural sway spectrum, pathological subjects do not. Precisely how the canceled frequencies are “chosen” by the postural control system is not known, but the minimization of muscular effort required for controlling the sway is perhaps one of the system’s major deciding factors. The PII which quantifies the postural performance by taking into account the two aforementioned indices (P and CT), was also calculated (Dumistrescu and Lacour, 2006; Bernard-Demanze et al., 2009) as follows: PII = ∑_x,y_ P(F1, F2, F3)/CT(F1, F2, F3). For healthy adults the PII is close to unity during the quiet stance task (Bernard-Demanze et al., 2009). This time-frequency analysis and associated parameters were obtained with the software PosturoPro (Framiral, Cannes, France).

#### Statistical analysis

In order to test Hypothesis 1 (a first glance at the sculpture has an immediate effect on posture), we run one-way ANOVAs with the main factor SCULPTURE (fixating the cross before viewing the sculpture *versus* fixating the sculpture). This analysis was performed on data from conditions 1 and 2.

In order to test Hypotheses 2 and 3 (exploration of the sculpture, with eye movements—Hypothesis 2—or during the *promenade*—Hypothesis 3, has an effect on posture), we run two-way ANOVAs with the main factors EYE MOVEMENTS (sculpture fixation *versus* eye navigation) and PROMENADE (before *versus* after visiting the exhibition). This analysis was performed on data from conditions 2–5.

*Post hoc* analyzes were conducted with the Fischer’s LSD test. The significance level was set at *p* < 0.05.

### RESULTS

#### The SCULPTURE effect: the power of medio-lateral body sway decreased at first glance

**FIGURE 2:**
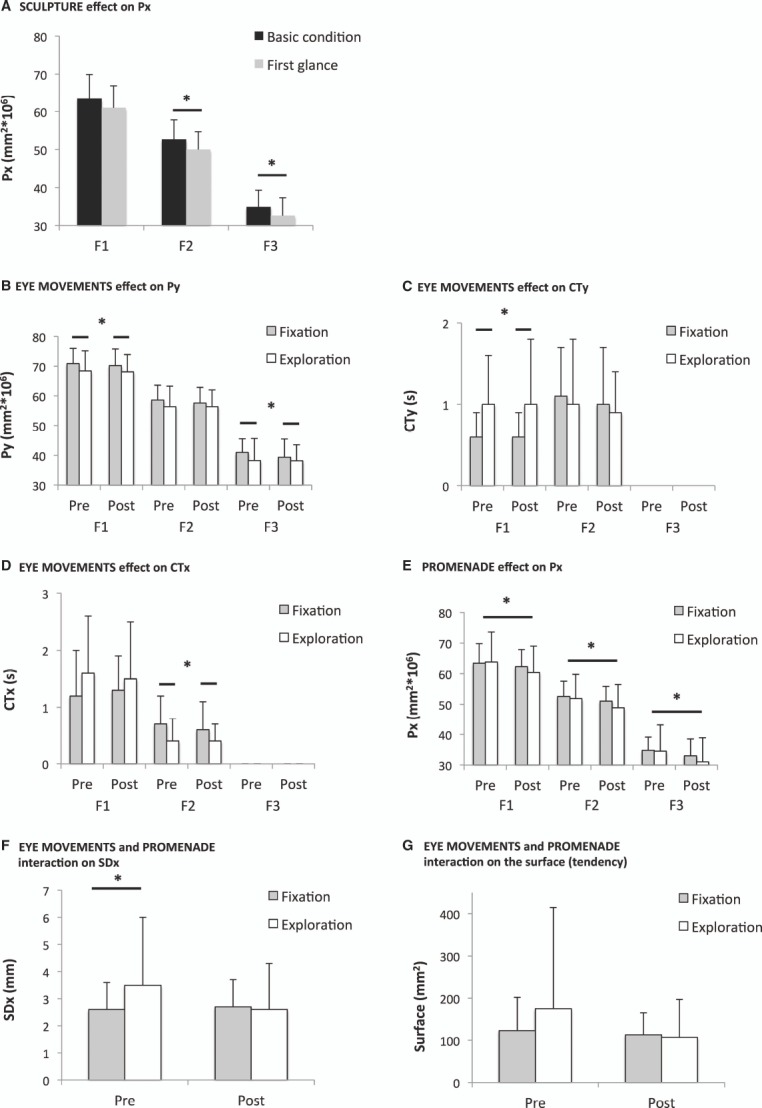
**Significant results of the Experiment 1.** Group mean and standard deviation. Legend: Basic condition: condition 1. First glance: condition 2. Pre: pre-promenade (conditions 2 and 3). Post: post-promenade (conditions 4 and 5). Fixation: fixating the sculpture (conditions 2 and 4). Exploration: eye navigation in depth along the sculpture’s transverse plan (conditions 3 and 5). F1: 0.05–0.5 Hz. F2: 0.5–1.5 Hz. F3: >1.5 Hz. The symbol * indicates significant *p* values. **(A)** Compared to the basic condition, fixating the sculpture significantly decreased the spectral power of medio-lateral sway (Px) for F2 and F3. **(B)** Navigating with eye movements along the sculpture significantly decreased the spectral power index of antero-posterior sway (Py) for F1 and F3. **(C)** Navigating with eye movements significantly increased the canceling time of antero-posterior sway (CTy) for F1. **(D)** Navigating with eye movements significantly decreased the canceling time of medio-lateral sway (CTx) for F2. **(E)** The *promenade* significantly decreased the spectral power of medio-lateral sway (Px) for all frequency bands, F1, F2, and F3. **(F)** Before the *promenade*, navigating with eye movements significantly increased the standard deviation of medio-lateral body sway (SDx); this was no longer the case after the *promenade.* **(G)** Before the *promenade*, navigating with eye movements tended to increase the surface of the CoP excursions (Surface); this was no longer the case after the *promenade.*

The one-way ANOVAs revealed that the SCULPTURE factor significantly decreased Px for *F*2 [*F*_(1,20)_ = 6.7; *p* < 0.02] and *F*3 [*F*_(1,20)_ = 5.0; *p* < 0.04; Figure 2A]. Thus, compared to the basic condition, a first glance of the laterally tilted sculptures caused a decrease in the power of the medio-lateral body sway. This is a subtle but significant, medio-lateral axis-specific, immediate effect.

#### The EYE MOVEMENT effects: exploring the sculpture with eye movements improved antero-posterior but deteriorate medio-lateral posture performance

The two-way ANOVAs showed that the EYE MOVEMENTS factor significantly (i) decreased Py for F1 [*F*_(1,21)_ = 6.5; *p* < 0.02] and F3 [*F*_(1,21)_ = 4.7; *p* < 0.04; Figure 2B]; (ii) increased CTy for F1 [*F*_(1,21)_ = 10.1; *p* < 0.005; Figure 2C]; and (iii) decreased CTx for F2 [*F*_(1,21)_ = 5.16; *p* < 0.03, Figure 2D]. Thus, compared to merely staring at a sculpture, moving the eyes across sculptures decreased the energy spent in controlling antero-posterior body sway and increased the CT of antero-posterior body sway, thereby improving postural control along the main axis of eye movements. This was accompanied by a decrease in the CT of medio-lateral body sway, i.e., a decreased postural performance along that perpendicular axis.

#### Interaction between EYE MOVEMENTS and PROMENADE: the promenade eliminated medio-lateral instability related to eye movements

The two-way ANOVAs showed a significant interaction between the PROMENADE factor and the EYE MOVEMENTS factor for SDx [*F*_(1,27)_ = 7.16; *p* = 0.0142; Figure 2F]. Before visiting *Promenade*, the subjects exhibited higher lateral body sway when they performed eye movements as compared to the fixation condition (p = 0.003); after visiting *Promenade*, this eye movement-related medio-lateral instability disappeared. In other words, after the *promenade* the medio-lateral body sway remained small indicating improved stability along this axis even when the eyes were moving. There was a similar tendency for the surface of body sway to increase in the eye moving condition before the *promenade* but to decrease in the eye moving condition after the *promenade* [*F*_(1,27)_ = 3.42; *p* = 0.07; Figure 2G]. Consequently, after the *promenade* the postural stability improved in the exploration condition, indicating optimal overall control with respect to both viewing conditions (fixation, exploration).

#### The PROMENADE effect: the power of the medio-lateral body sway decreased after the visit

The two-way ANOVAs showed that the PROMENADE factor significantly decreased Px for F1 [*F*_(1,21)_ = 5.9; *p* < 0.02], F2 [*F*_(1,21)_ = 9.5; *p* < 0.01], and F3 [*F*_(1,21)_ = 11.2; *p* = 0.003; Figure 2E]. Thus the *promenade* around the laterally tilted sculptures resulted in a substantial decrease in the power of medio-lateral body sway across all frequency ranges.

## EXPERIMENT 2: SUBJECTIVE VISUAL VERTICAL

### MATERIALS AND METHODS

#### The artwork

The artwork was the same as in Experiment 1.

#### Participants

Fourteen new participants (29.5 ± 9.0 years) volunteered to take part in this second study.

#### Procedure

In Experiment 2, participants were invited to visit the exhibition under the same conditions as in Experiment 1. The participants’ SVV was examined before and after visiting the exhibition with a dedicated device (Framiral, Cannes, France). The SVV was determined by inviting participants to stand in a dark room and adjust a luminous line to what they considered to be the earth-vertical axis. This test was carried out in an adjacent room of the museum where complete darkness was easily achieved. The reported error measures were absolute. To our knowledge, learning of the SVV task when examinations are performed tens of minutes apart has never been reported. Thus, any change in SVV errors can be reasonably attributed to the *promenade* itself (i.e., visiting the *Promenade* sculpture while listening to the audio-guide).

#### Statistical analysis

In order to test Hypothesis 4 (the *promenade* has an effect on SVV), a one-way ANOVA was run on the SVV error data with the PROMENADE factor (before *versus* after visiting the exhibition).

### RESULTS

**FIGURE 3:**
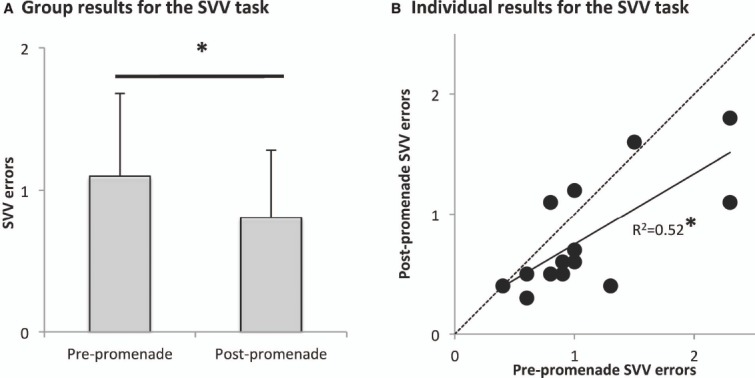
**Significant results of the Experiment 2.** The symbol * indicates significant *p* values. **(A)** Group mean and standard deviation of the SVV errors; the *promenade* significantly decreased the SVV errors. **(B)** Individual SVV errors before and after the promenade are correlated; most of subjects have smaller errors after the *promenade* than before.

Figure 3A indicates group mean and standard deviations of SVV errors before and after the promenade. The one-way ANOVA showed that the PROMENADE factor significantly decreased the SVV errors [*F*_(1,13)_ = 8.81; *p* = 0.01].

In addition, examination of Figure 3B displaying individual SVV errors before and after the promenade showed that: (i) there was a correlation between pre- and post-*promenade* measures (which was confirmed by a significant Pearson correlation: R^2^ = 0.52; *p* = 0.0036) and (ii) the *promenade* improved the SVV for 10 participants, decreased it for three participants and did not change it for one participant. Thus, although the performance of individual participants before and after the *promenade* was correlated, the *promenade* induced a small but significant improvement of the SVV, found for the large majority of the participants (71%).

## DISCUSSION

### FIRST GLANCE: THE IMMEDIATE EFFECT

The first glance at these laterally tilted monumental sculptures causes a subtle decrease of the spectral power of medio-lateral body sway. We note the immediacy and specificity of this effect on body sway which is shown to occur along the same anatomical axis as the geometric axis about which the sculpture itself is tilted. This observation is in keeping with our previous posturographic analyses of paintings (Kapoula et al., 2011; Kapoula and Gaertner, 2014), which also revealed the immediate effects of paintings on postural control. Indeed, the present study further corroborates these previous findings while at the same time extending its reach for the first time to include statuary works. The observed immediacy and specificity indicate the impact, however, subtle, of artwork in general on human physiology.

### EFFECTS OF EYE MOVEMENTS

Testing the effects of visual exploration on postural control was primarily achieved by comparing fixation of the sculpture to eye movement navigation in depth along the sculptures’ transverse plane. As explained in Section “Materials and Methods,” the eye movements involved convergence and divergence eye movements, which were most likely combined with lateral and/or vertical saccades. Although eye movement recording was not possible, inspection of the subjects’ eyes by the experienced eye movement investigators clearly helped to confirm the occurrence of the aforementioned naturally combined eye movements. Subjects made approximately 15 back and forth excursions fixating the five sculptures successively one after the other. Such combined eye movements are in fact the most natural eye movements we make in real life to explore our 3D environment. The impact of such movements on postural control has not yet been studied. Instead some studies from our own research group have examined the effect of saccades and the effect of vergence along the median plane separately, using basic experimental stimuli such as fixating a cross or a laser dot that moves in depth. Saccades alone were not found to compromise postural stability and in fact some benefits were even observed (Rey et al., 2008). In so far as vergence eye movements were concerned, a clear beneficial effect was reported in both healthy subjects and adult patients (Kapoula et al., 2013). The effects observed in the present study relative to combined saccade–vergence eye movements along the sculptures also indicate an improvement in the time-frequency domain along the antero-posterior, depth axis. The spectral power decreased and the cancellation time increased, which indicate longer time periods of optimal control. Whether or not this effect is purely physiologic and could be observed any time we make such eye movements even when directing our gaze at stimuli which were not necessarily designed with an artistic or aesthetic end in mind (e.g., a laser dot moving in depth with some lateral or vertical component) is not known. It is likely that this would be the case and we fully expect the sculptures to accentuate effects of this kind; however, this hypothesis requires further investigation. It is important to note that eye navigation in depth along the sculptures’ transverse plane improved body sway control in precisely this antero-posterior direction. Another physiologic correlate of interest was the decrease in cancellation time of medio-lateral body sway which indicates that the optimization in the antero-posterior depth along the predominant eye movement axis was obtained at the expense of the lateral axis.

In summary, whether or not these observations are specific to sculptures, this study demonstrates that moving the eyes across sculptures that are laterally inclined and aligned in depth modifies the control of body sway. Navigating Serra’s *Promenade* using solely ones eyes can thus be considered an active visual process, as opposed to a purely static, passive and receptive one; a *promenade* of the eyes so to speak, which calls the body into action during the dynamic control of quiet stance.

### INTERACTION BETWEEN EYE MOVEMENTS AND THE PROMENADE

Another major finding concerns the interaction between eye movements and the *promenade* around and alongside the sculptures. Visiting the sculptures for approximately 1 h with an audio-guide was a multisensory, physiologic and aesthetic experience. Indeed, at the purely physiologic level, while the observers walk around and alongside the sculptures, they generate a large bank of visual, vestibular, and sensorimotor data, which is integrated into and associated with the aesthetic experience. Walking involves canal and otolithic stimulation, compensatory vestibularly generated eye movements as well as somatosensory and visual experiences (the optic flow contingent on one’s body motion as one navigates into the sculpted space of the artwork). Walking as a motor behavior is essentially organized around the vertical axis and the body’s orientation and stabilization in space depend on the integration of all of these signals (Montgomery, 1985; Hegeman et al., 2007). This sensorimotor experience combined with listening to the exhibit’s handheld audio-guide involves physiologic, cognitive, and emotional processes whose combined effects are apparently quite lasting. Indeed, the results indicate that the partial destabilization in body control along the medio-lateral axis triggered during simulated visual navigation into the sculpted environment was ultimately abolished after engaging in the full-body navigation, i.e., the *promenade.* After the *promenade*, the medio-lateral body sway was under much better control even when the eyes were moving as it became similar to the sway under the fixating condition. As previously stated, we attribute this effect to the physiologic experience provided by the *promenade* and its lasting beneficial effects. In other words, exposure to the sculptures would seem to have acted as a physiologic “training” stimulus, not unlike the training modalities used in vestibular rehabilitation techniques. This interpretation is further corroborated by overall improvement of postural control and SVV to be discussed below.

### THE EFFECT OF THE PROMENADE

The promenade decreased the spectral power of medio-lateral body sway. This was a major, subtle lasting effect that was significant for all frequency ranges. Again, this beneficial effect concerned the medio-lateral body sway, which was presumably related to the medio-lateral tilt of the sculptures. It has been demonstrated in laboratory studies that exposing healthy subjects to a tilted visual reference can cause a deviation in their body’s position (Isableu et al., 1997). Presumably, the tilted sculptures initially produced such an effect resulting in a subsequent self-correcting and compensatory postural response designed to keep the body in an upright vertical position while ambulating and navigating in the sculpted environment of the artwork. All of these self-corrections might have also improved orthostatic posture during quiet stance. Postural control during quiet stance and postural control while ambulating are highly interdependent (Bent et al., 2002). A decrease in the spectral power is indicative of improved control over medio-lateral body sway which is presumably mediated by the prolonged sensorimotor, vestibular, and visual experience of the observer’s *promenade* around and alongside the tilted sculptures.

### IMPROVEMENT OF THE SUBJECTIVE VISUAL VERTICAL

Healthy subjects are able to perceive verticality with considerable accuracy and this faculty is dependent on inputs from visual, vestibular, proprioceptive, and somatosensory systems (Friedmann, 1970; Pavlou et al., 2003). The perception of verticality also depends on a functioning central nervous system (Yelnik et al., 2002). The otolithic organs in the vestibular system sense gravity and the utricle and saccule contribute to a sense of verticality. Following injury to the otoliths or to the nerve that transmits signals from the otoliths and other parts of the ear to the brain, verticality judgments may be altered. Visual influences on verticality may be measured by putting a frame around a rectilinear bar. Alterations in the angle that the frame forms relative to the bar may disturb a person’s judgment of the bar’s verticality (e.g., Gueguen et al., 2012). Subjects with vestibular lesions may orient the bar such that it deviates from true vertical by as much as 10^°^ (Vibert et al., 1999; Gomez Garcia and Jauregui-Renaud, 2003).

In this study, we found that the *promenade* was associated with a statistically significant reduction in errors in the estimation of SVV. Prior to the participants’ *promenade*, the mean value for SVV was small, i.e., 1.1^°^ which is within normal values (<2^°^, see Friedmann, 1970). Immediately following their *promenade*, this error rate continued to decrease falling to a mere 0.8^°^ which reflects a performance of considerable accuracy. We attribute this ameliorative effect to the same mechanism responsible for the postural results, i.e., to the vestibular–visual signals generated while walking and interacting with the artwork. Indeed, it is known that both postural control and one’s perception of SVV rely on the integration of vestibular, somatosensory, proprioceptive, and visual signals (Friedmann, 1970; Pavlou et al., 2003; Blumle et al., 2006). As mentioned above, walking around the sculptures stimulates otolith, visual, proprioceptive, and somatosensory signals and these signals are ultimately integrated, however, imperfectly. It therefore follows that an improvement in one’s estimation of SVV could also be due to a concomitant improvement in one’s ability to effectively integrate these diverse signals. Whether or not, in addition to relying on common signals, posture control and SVV may directly influence each other (see, e.g., the model suggested by Barra et al., 2012) in an experimental set-up similar to ours remains to be investigated in future studies.

### ARTWORKS ILLUSTRATE THEORIES OF EMBODIED COGNITION

Artworks in general are excellent opportunities to illustrate the theories of embodied cognition. Wilson (2002) distinguished and evaluated six main propositions that are embedded within the concept of embodied cognition. By definition, visiting a exhibition, e.g., walking around sculptures, illustrates that human cognition is a situated activity (proposition #1): we do not merely get informed about an artist and her/his ideas but we go experience her/his artwork. Our findings concerning the artwork of Richard Serra’s is specifically adding evidence to other propositions. For instance, the statement that the environment is part of the cognitive system (proposition #4) can be understood within the framework of a complex interaction between two minds, the artist’s and the visitor’s, mediated by their bodies and the environment. Indeed, the artist creates the physical artwork, which influences the body of the observer (her/his physiology, including posture and sense of verticality), which in turn probably influences her/his cognition. Our experiment specifically probes the link between the physical artwork and the body of the observer, while future studies might address other links, e.g., between the body of the observer and her/his subjective appreciation of the artwork. Serra’s sculpture also allowed us to test the proposition that cognition is for action (proposition #5). Indeed, as the mere vision of a mug is an affordance and triggers a motor activation related to the action of seizing, the experience of tilted sculptures probably caused compensatory movements to adapt body balance to such unusual environment, at the origin of the effects described in the present study. We hope the role of body physiology in cognition will be further demonstrated in future studies, e.g., exploring whether remembering an artwork triggers similar postural effects than when actually visiting the exhibition, which would contribute to explore whether off-line cognition is body-based (proposition #6). Thus, we believe that measuring body physiology in various cognitive situations, including when interacting artwork, is importantly contributing to theories of embodied cognition.

## CONCLUSION

Visiting this exhibition involved emotional (e.g., facing the monumental sculpture within the Grand Palais’s neve), cognitive (e.g., listening to the audio-guide), and physiologic (e.g., observing this stimuli strongly playing with sense of depth and verticality, exploring it with combined eye movements and deambulation around it) processes. Ambulating within the sculpted environment of Serra’s monumental statuary work, *Promenade*, contributed, according to the evidence marshaled in the present study, to subtle improvements in the participants’ postural control as well as in their capacity to accurately judge the SVV. Such improvements in postural control and sense of verticality might be related to the fact that such aesthetic stimulus is physiologically challenging for the human body. Such findings might be of interest to the field of Art Therapy. Notwithstanding cognitive and emotional evaluations, aesthetic experiences doubtless involve the body in its entirety, including our sense of gravity and verticality. The participants came out of the exhibition with an improved sense of verticality; this can contribute to the elaboration of aesthetic experience, which is existential and contributes to personal integrity according to philosophers (Funch, 1997).

### Conflict of Interest Statement

The authors declare that the research was conducted in the absence of any commercial or financial relationships that could be construed as a potential conflict of interest.
